# EphA2 signaling is impacted by carcinoembryonic antigen cell adhesion molecule 1-L expression in colorectal cancer liver metastasis in a cell context-dependent manner

**DOI:** 10.18632/oncotarget.22236

**Published:** 2017-11-01

**Authors:** Azadeh Arabzadeh, Kevin McGregor, Valérie Breton, Lauren Van Der Kraak, Uri David Akavia, Celia M.T. Greenwood, Nicole Beauchemin

**Affiliations:** ^1^ Goodman Cancer Research Centre, McGill University, Montreal, QC, Canada; ^2^ Department of Epidemiology, Biostatistics & Occupational Health, McGill University, Montreal, QC, Canada; ^3^ Department of Biochemistry, McGill University, Montreal, QC, Canada; ^4^ Lady Davis Institute, Jewish General Hospital, Montreal, QC, Canada; ^5^ Departments of Oncology and Human Genetics, McGill University, Montreal, QC, Canada; ^6^ Departments of Medicine and Oncology, McGill University, Montreal, QC, Canada

**Keywords:** CEACAM1, CEA, CEACAM6, EPHA2, liver metastasis

## Abstract

We have shown that carcinoembryonic antigen cell adhesion molecule 1 long isoform (CEACAM1-L) expression in MC38 metastatic colorectal cancer (CRC) cells results in liver metastasis inhibition via CCL2 and STAT3 signaling. But other molecular mechanisms orchestrating CEACAM1-L-mediated metastasis inhibition remain to be defined. We screened a panel of mouse and human CRC cells and evaluated their metastatic outcome after CEACAM1 overexpression or downregulation. An unbiased transcript profiling and a phospho-receptor tyrosine kinase screen comparing MC38 CEACAM1-L-expressing and non-expressing (CT) CRC cells revealed reduced ephrin type-A receptor 2 (EPHA2) expression and activity. An EPHA2-specific inhibitor reduced EPHA2 downstream signaling in CT cells similar to that in CEACAM1-L cells with decreased proliferation and migration. Human CRC patients exhibiting high *CEACAM1* in combination with low *EPHA2* expression benefited from longer time to first recurrence/metastasis compared to those with high *EPHA2* expression. With the added interaction of *CEACAM6*, we denoted that *CEACAM1* high- and *EPHA2* low-expressing patient samples with lower *CEACAM6* expression also exhibited a longer time to first recurrence/metastasis. In HT29 human CRC cells, down-regulation of CEACAM1 along with CEA and CEACAM6 up-regulation led to higher metastatic burden. Overall, CEACAM1-L expression in poorly differentiated CRC can inhibit liver metastasis through cell context-dependent EPHA2-mediated signaling. However, CEACAM1’s role should be considered in the presence of other CEACAM family members.

## INTRODUCTION

Liver metastasis of colorectal cancer (CRC) occurs in approximately 30% of CRC patients and is a major cause of CRC related mortality [1]. While improvements in surgical techniques, chemotherapeutic regimens and the availability of anti-EGFR- and anti-VEGFR2-targeted therapies have contributed to longer survival and better quality of life for CRC metastatic patients, further research is needed to identify novel metastatic targets for therapeutic intervention. Carcino embryonic antigen cell adhesion molecule 1 (CEACAM1), a member of the *CEA* gene family, is a cell adhesion molecule known to be associated with CRC tumor development and metastasis [2]. Numerous human and murine CEACAM1 splice variants have been identified that differ with respect to the expression of either a short (S) or a long (L) cytoplasmic domain. CEACAM1-L becomes Tyr phosphorylated on its two cytosolic Tyr residues within its immunoreceptor tyrosine inhibition motifs (ITIMs) by several activated receptor tyrosine kinases (RTKs) or SRC-like kinases leading to binding of the SHP-1 or -2 Tyr phosphatase [2]. CEACAM1-L is multifunctional and acts as a negative regulator of many signaling pathways [3] involved in intercellular adhesion regulation [4], insulin and lipid metabolism [5, 6], angiogenesis [7], innate and adaptive immune responses [8–10] and microbial and viral pathogen interactions [3].

In tumor development, CEACAM1 plays a paradoxical role. CEACAM1 down-regulation is associated with initiation and early development of several solid tumors including CRC [2, 11]. However, CEACAM1 behaves as an oncogene in aggressive cancers. CEACAM1-L expression mediates tumor development within tumor cells directly (colon [12], melanoma [13], non-small-cell lung cancer [14], thyroid [15], gastric [16]) as well as indirectly via cells in the stromal compartment (endothelial cells [7, 17], CD11b^+^Gr1^+^ immature myeloid cells [18, 19], matrix metalloproteinase 9-positive leukocytes [20], tumor-associated macrophages [21] and activated T cells [8]).

Ieda and colleagues reported that CEACAM1-L dominance over CEACAM1-S in human CRC corresponds to increased lymph node and hematogenous metastasis, in addition to shorter patient survival [12]. However, our studies in murine poorly differentiated MC38 CRC cells demonstrated reduced liver metastatic burden with increased CEACAM1-L expression, in part due to diminished levels of CCL2 and STAT3 activity [22]. Furthermore, we showed that patients exhibiting high *CEACAM1* expression along with a signature of inflammation- and STAT3-regulated genes demonstrate improved 10-year overall survival [22].

To determine whether CEACAM1-L produces similar metastasis corollary in other CRC cells, we investigated a large panel of human and mouse CRC cells presenting unique mutations and expression of different CEACAM family members. We show here that up- or down-regulation of CEACAM1 does not change metastasis outcome in all cases, except in HT29 cells. Notably, HT29 cells have a similar *KRAS* and *SMAD4* mutational status as MC38 cells, despite being different with respect to CEA and CEACAM6 expression. Moreover, knockdown of CEACAM1 in HT29 cells led to up-regulation of both CEA and CEACAM6 that altogether increased liver metastatic burden.

To define other CEACAM1-L-elicited networks regulating liver metastasis, we performed unbiased transcriptome and phospho-receptor tyrosine kinase (RTK) screens of the MC38 cells that do (MC38-CC1-L) or don’t (MC38-CT) express CEACAM1-L. Gene expression profiling and phospho-RTK screens revealed that the EPHA2 receptor, a member of the EPH family of receptors [23], is down-regulated in MC38-CC1-L cells both at the transcriptional and activity levels. In human CRC patients, increased EPHA2 expression levels are positively correlated with cancer progression and liver metastasis [24–26]. We demonstrate herein that CEACAM1-L expression modulates the expression and activity of the EPHA2 receptor in a cell context-dependent manner and that inhibition of EPHA2-mediated signaling also inhibits metastasis. Furthermore, bioinformatics analyses of TCGA CRC patient cohorts confirm that a signature of high *CEACAM1*/low *EPHA2*/low *CEACAM6* gene expression corresponds to significantly longer time to first recurrence/metastasis for CRC patients. Therefore, CEACAM1, CEACAM6 and EPHA2 represent additional actionable targets to increase overall survival in cohorts of patients with liver metastasis produced by poorly differentiated CRC.

## RESULTS

### CEACAM1-L-mediated metastasis inhibition is dependent on CRC cell context

**Figure 1:**
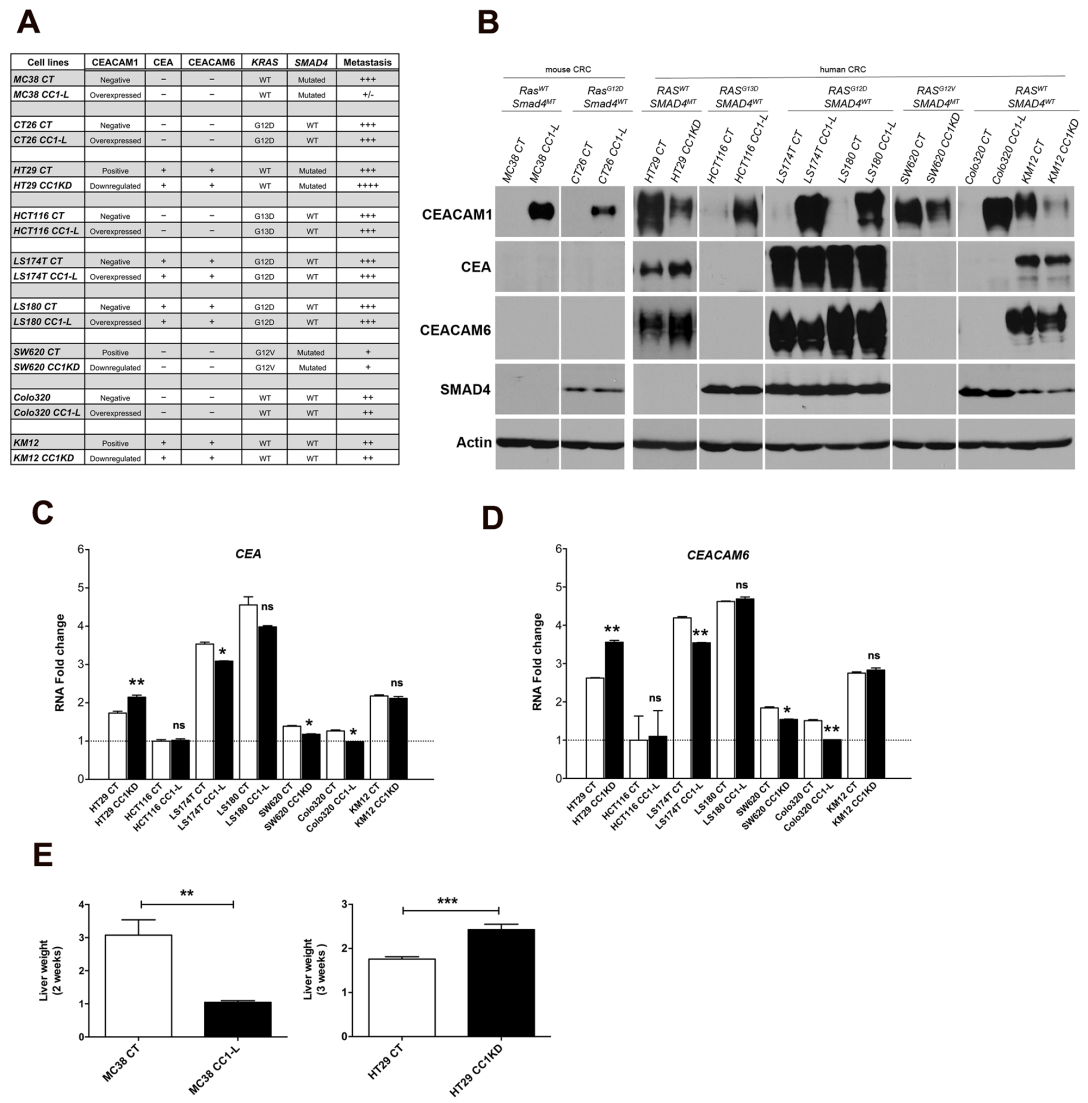
Screening of mouse and human CRC cells with respect to mutational background, expression of CEA and CEACAM6, and *in vivo* metastasis **(A)** Mouse and human CRC cells were investigated for their mutational status of *KRAS* and *SMAD4* (using DNA sequencing data), expression of pro-metastatic CEACAM molecules (CEA and CEACAM6) (based on results of protein levels) and their metastatic potential *in vivo*. A summary of these results is tabulated. Under metastasis column, +/-, +, ++, +++, ++++ denote arbitrary values based on increase in basal liver weight, corresponding to <10%, 20%, 30%, 50-60%, and >80% increases, respectively. **(B)** Protein expression of CEACAM1, CEA, CEACAM6, SMAD4 and actin was evaluated by immunoblotting CRC cell lysate proteins. Cell lysates are categorized based on their *KRAS* and *SMAD4* mutational status. **(C-D)** *CEA* and *CEACAM6* RNA expression from human CRC cells was performed by Q-PCR and normalized to *RPLP* expression. All fold change (FC) values were normalized to the FC value (set at 1) of lowest *CEA*- and *CEACAM6*-expressing cells, namely HCT116 cells. Note that mouse cells do not intrinsically express the *CEA* and *CEACAM6* genes and no Q-PCR was performed for these. **(E)** Evaluation of *in vivo* liver metastasis development by murine (MC38-CT and -CC1-L) or human (HT29-CT and -CC1KD) CRC cells. C57BL/6 mice were injected with MC38 cells and monitored for 2 weeks, while SCID Beige mice were injected with HT29 transfectants and euthanized after 3 weeks. Liver weights were measured and represent metastatic burden. Student two-tailed *t* or ANOVA with Bonferroni correction tests were performed as appropriate to determine significance (ns, *P* > 0.05; ^*^, *P* < 0.05; ^**^, *P* < 0.01; ^***^, *P* < 0.001). Data are presented as means ± SEM with n = at least 2 independent sets of experiments. For *in vivo* studies a minimum of 10 mice per group were used.

We have previously shown that CEACAM1-L expression in poorly differentiated murine MC38 CRC metastatic cells results in an approximate 80% reduction in liver metastatic tumor burden following intrasplenic injection in C57BL/6 mice [22]. This is in part due to compromised STAT3 activity which results in reduced CCL2 chemokine expression and decreased signaling through its CCR2 receptor. To determine whether this is a general phenomenon applying to other CRC cells, we investigated a panel of mouse (MC38, CT26) and human (HT29, HCT116, LS174T, LS180, SW620, Colo320, KM12) metastatic CRC cells. As summarized in Figure 1A, these cells show different status of *KRAS* and *SMAD4* mutational status, two genetic mutations frequently observed in CRC patients [27–33], as well as varying levels of CEACAM1 expression and that of two other well-known pro-metastatic CEACAM family members namely CEA and CEACAM6 [2]. To examine how CEACAM1-L affected development of CRC liver metastasis, we either overexpressed or down-regulated CEACAM1 using appropriate vectors (Figure 1A). Cells exhibiting CEACAM1 knockdown (CC1KD: HT29, SW620 and KM12) or with its overexpression (CC1-L: MC38, CT26, HCT116, LS174T, LS180, Colo320) were compared to their control counterparts (CT) in all assays. Mouse CRC cells do not normally express any of glycosylphosphatidylinositol (GPI)-anchored CEACAM proteins including CEA and CEACAM6 (Figure 1A and 1B), as these genes do not exist in the murine genome [34]. Among human CRC cells, HT29, LS174T, LS180 and KM12 expressed both molecules abundantly either at the protein level (Figure 1B) or at the transcriptional level (Figure 1C-1D). HCT116 cells had no CEA and CEACAM6 at mRNA or protein levels, whereas SW620 and Colo320 cells exhibited low mRNAs but undetectable CEA and CEACAM6 protein (Figure 1B-1D). Overexpression or knockdown of CEACAM1 did not appreciably change the levels of the other two CEACAM proteins in LS180 or KM12 cells, with only modest reductions in LS174T cells. However, both CEA and CEACAM6 proteins were notably up-regulated in HT29 cells upon CEACAM1 knock-down (Figure 1B-1D).

*In vivo* experimental metastatic assays demonstrated that except in the case of MC38 cells [22] and HT29 cells (Figure 1E), CEACAM1-L did not change the outcome of metastasis in other CRC cells tested (Supplementary Figure 1). Notably, HT29 cells have a similar *KRAS* and *SMAD4* mutational status as MC38 cells, despite being different with respect to presence of both CEA and CEACAM6. In this context, reduced CEACAM1 possibly in combination with increased CEA and CEACAM6 in HT29 cells contributed to increased liver metastatic burden (Figure 1E). This suggests that co-regulation of 3 CEACAM family members may have an additive effect on the outcome of metastasis and modification of one protein likely impacts on the expression of other CEACAM family members. Altogether, these results suggest that the fate of metastatic CRC cells may be affected by CEACAM1-L in a cell context-dependent manner, where mutational cell background or presence/absence of other CEACAM family members in human CRC cells may override the reported anti-metastatic effect of CEACAM1-L.

### The EPHA2 receptor exhibits decreased expression and activity in MC38-CC1-L cells

**Figure 2:**
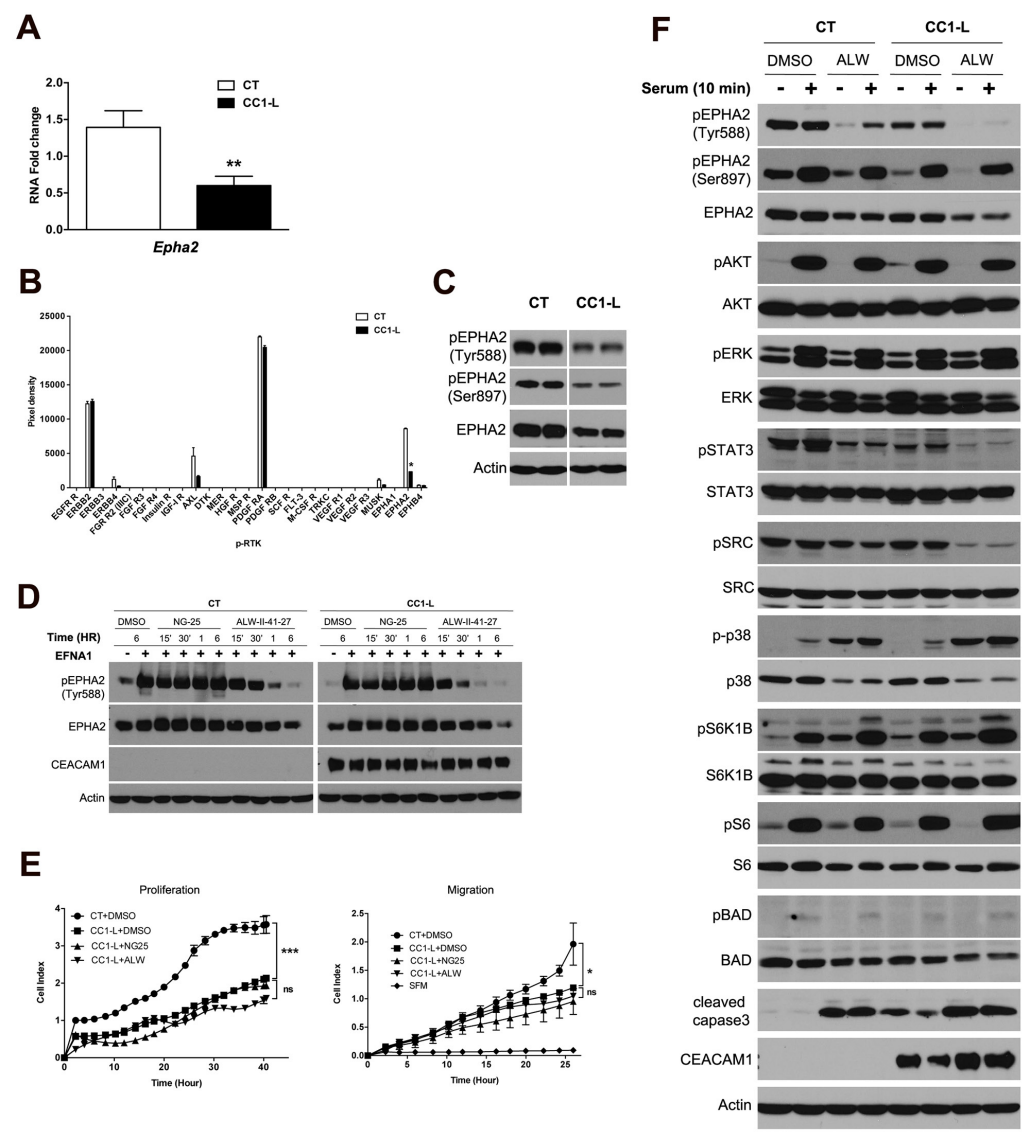
Contribution of the EPHA2 receptor signaling to CC1-L-mediated metastasis inhibition in MC38 cells **(A)** Validation of *Epha2* expression by Q-PCR, normalized to *Psmb6* expression for MC38-CT vs -CC1-L. **(B)** A phospho-RTK array was performed on protein lysates from MC38-CT or -CC1-L cells as described in Materials and Methods. Kinase phosphorylation was quantified and those showing prominent levels of activity were subjected to further validation by immunoblotting of new batches of lysates (2C and Supplementary Figure 3A-3B). **(C)** EPHA2 receptor phosphorylation (pTyr588 and pSer897) and expression in MC38-CT or -CC1-L cell lysates were measured by immunoblotting. Actin was used as the loading control. **(D)** MC38-CT or -CC1-L cells were treated with 1 μM of DMSO, or the non-specific NG-25 or specific ALW-II-41-27 EPHA2 receptor inhibitor for indicated periods of time, followed by stimulation with EFNA1-Fc (2 μg/ml) in the last 15 min of treatments. Protein lysates were prepared and subjected to immunoblotting using antibodies to determine the effect of kinase inhibitors on EPHA2 activation. Actin was used as the loading control. **(E)** Effect of two receptor Tyr kinase inhibitors, differing in their activity against EPHA2, was examined through proliferation and migration assays. MC38-CT or -CC1-L cells were treated with 1 μM ALW-II-41-27, NG-25 or DMSO for 2 h after which they were set up for proliferation and migration assays. Migration took place in the presence of 10% serum as chemoattractant, while serum-free medium (SFM) was used as negative chemotaxis control. **(F)** MC38-CT or -CC1-L cells were treated with 1 μM DMSO or ALW-II-41-27 for 6 h, followed by serum starvation overnight. Cells were then stimulated or not with 10% serum for 10 min before lysis. Expression levels of various signaling proteins were evaluated by immunoblotting using total protein and phospho-protein antibodies as indicated. Student two-tailed *t* or ANOVA with Bonferroni correction tests were performed as appropriate to determine significance (ns, *P* > 0.05; ^*^, *P* < 0.05; ^**^, *P* < 0.01; ^***^, *P* < 0.001). Data are presented as means ± SEM with n = at least 2 independent sets of experiments.

To identify other targets involved in CEACAM1-L-mediated CRC liver metastasis suppression, we first profiled the mRNA expression of MC38-CT and -CC1-L cells using an unbiased transcript microarray. Filtering through Ingenuity pathways identified five clusters with differentially expressed genes showing over-representation in metabolic processes, cellular transport and communications, and cellular localization pathways (Supplementary Figure 2). Differentially expressed genes in MC38-CC1-L relative to -CT cells were selected based on statistically significant F-values (*P*<0.05) and False Discovery Rates (<0.05) and were further validated using qPCR. Genes differentially over-expressed in MC38-CC1-L relative to -CT cell microarray (GEO dataset, accession # GSE73208) included decorin (*Dcn*), a member of the small leucine-rich proteoglycan gene family, that was the most over-expressed RNA in the MC38-CC1L cells [35]. In CRC tissue and cell lines, *Dcn* is usually down-regulated [36] and *Dcn*^-/-^ mice fed a high-fat diet develop spontaneous intestinal tumors through disruption of enterocyte maturation [37]. Furthermore, DCN binds to E-cadherin and mediates E-cadherin protein stability that results in inhibition of CRC HCT116 cell migration [38]. Other up-regulated transcripts included *Bmp4* and *Tgfβr2*, involved in bone morphogenic protein (BMP) and transforming growth factor β (TGFβ) signaling, respectively, as well as alpha B crystallin (*Cryab*) implicated in CRC distant metastasis [39] and bone marrow stromal cell antigen 2 (*Bst2*) overexpressed in breast cancer bone metastasis [40]. Examining the down-regulated genes in this microarray, *Epha2* corresponded to one of the down-regulated genes in the microarray analysis (Figure 2A; 50-70% reduction). CEACAM1-L expression affects many cellular processes through its co-receptor function with a number of receptor Tyr kinases [2] and given that EPHA2, a member of the EPH family of receptors [23], is a receptor Tyr kinase (RTK), we further investigated which other RTKs were activated in MC38-CT cells and reduced in MC38-CC1-L cells by applying cell lysates to a mouse phospho-RTK array. Seven RTKs were significantly expressed and activated in both cell lines (Figure 2B) with EPHA2 being the only RTK differentially activated between the two cell lines. Reduced activity and expression of EPHA2 was confirmed by immunodetection of pEPHA2-Tyr588 and pEPHA2-Ser897 in MC38-CC1-L versus MC38-CT cells (Figure 2C) which supports previously published literature showing correlation between increased EPHA2 expression levels and CRC cancer progression/liver metastasis [24, 25]. No noteworthy differences in ERBB2 activity in basal and EGF-stimulated MC38-CT and -CC1-L cells were observed (Supplementary Figure 3A). Similarly, PDGFRA expression levels and basal activity were unchanged in these cells (Supplementary Figure 3B). While AXL activity appeared lower in MC38-CC1-L cells compared to MC38-CT cells (Figure 2B), we observed no changes in the AXL receptor phosphorylation or AXL-mediated downstream signaling to STAT3 and AKT upon treatment with its GAS6 ligand (data not shown).

EPHA2 activity and expression were then investigated in other mouse and human CRC cells (Supplementary Figure 3C). CRC cells expressed varying levels of the EPHA2 receptor along with different degrees of Tyr588 phosphorylation (tyrosine controlling kinase activity) and Ser897 phosphorylation (ligand-independent serine). Among human CRC cells, only HT29 cells exhibited increased Tyr and Ser phosphorylation upon CEACAM1 knockdown (Supplementary Figure 3C) that mirrors reductions in EPHA2 pTyr and pSer upon CEACAM1-L over-expression in MC38 cells (Figure 2C). These results suggest that CEACAM1-L impacts on expression and/or activity of EPHA2 receptor Tyr kinase in some CRC cells. Whether or not heterogeneity of a tumor or different mutations acquired by cancer cells in CRC patients can modulate CEACAM1’s effect on EPHA2 activity has yet to be investigated.

### Inhibition of EPHA2 kinase suppresses MC38 cell growth and impacts downstream signaling

To confirm that EPHA2 signaling regulated CRC metastasis, MC38-CT and -CC1-L cells were first stimulated with ephrin A1 (EFNA1), an EPHA2 receptor ligand [23] and then downstream signaling was evaluated by immunoblotting of various proteins regulating several biological processes. EFNA1-EPHA2 binding resulted in increased EPHA2 Tyr588 phosphorylation activity in MC38-CT and -CC1-L cells followed by receptor internalization and degradation (Supplementary Figure 4). As noticed previously, MC38-CC1-L cells expressed less EPHA2 receptor than -CT cells and showed reduced EPHA2 Tyr588 phosphorylation activity relative to CT cells 2 h post-stimulation (Supplementary Figure 4; *P* < 0.05; ANOVA for groups *P* < 0.0001). Some signaling proteins downstream of EPHA2 stimulation in CC1-L cells showed slower deactivation kinetics than in CT cells such as pSTAT3 and pERK. Some pathways (e.g. p38) were deactivated faster, whereas the SRC pathway was indeed increased and sustained in CC1-L cells (Supplementary Figure 4). The AKT pathway was the most negatively affected with the first wave of phosphorylation being almost abolished in CC1-L cells, whereas the second wave peaked with the same intensity as in CT cells 30 min post-stimulation (Supplementary Figure 4). These results suggest that CEACAM1-L impacts both positively and negatively on various EPHA2 downstream pathways upon EFNA1 stimulation.

We then tested on MC38 cells several EPHA2 Tyr kinase inhibitory compounds. ALW-II-41-27 is a small molecule inhibitor binding to and inhibiting EPHA2 kinase activity with high potency [41–44]. NG-25, a compound with similar structure and profile of kinase targets with exception of EPHA2 was used as a control. In MC38-CT and -CC1-L cells, 1μM ALW-II-41-27 impaired EFNA1-induced EPHA2 Tyr phosphorylation within 30 min and continued to do so through 6 h of treatment (Figure 2D). However, NG-25 at the same concentration did not exhibit any effect on EPHA2 Tyr phosphorylation in either cell lines (Figure 2D). MC38-CT cells treated with ALW-II-41-27 inhibitor exhibited significant reductions in proliferation and migration (Supplementary Figure 5A and 5B: ns, *P* > 0.05 relative to DMSO or NG-25 treatments; ^*^, *P* < 0.05 or ^***^, *P* < 0.001 relative to DMSO vs ALW-II-41-27 treatments). MC38-CC1-L cells with reduction of EPHA2 expression and activity were less sensitive to the inhibitory effects of ALW-II-41-27 on proliferation and particularly migration (Figure 2E: ns, *P* > 0.05 relative to DMSO vs ALW-II-41-27 treatments in CC1-L cells; ^*^, *P* < 0.05 or ^***^, *P* < 0.001 relative to DMSO treatments in CT vs CC1-L cells). As depicted in the graphs, NG-25 had nearly no effect on these processes. These results suggest that EPHA2 is a potential target to inhibit metastasis of MC38 cells.

We next collected lysates from serum-starved MC38-CT and -CC1-L cells treated for 6 h with either ALW-II-41-27 or DMSO, in order to assess intracellular consequences of EPHA2 inhibitor targeting. Immunoblotting analyses revealed reductions in both the basal and serum-stimulated phosphorylation of STAT3 and SRC (regulators of cell proliferation, migration and invasion) as well as increases in phosphorylation of p38 and cleaved caspase3 (regulators of migration and cell death) at baseline and upon serum stimulation (Figure 2F). Such modifications were much more pronounced in MC38-CC1-L cells (see Supplementary Table 4 for quantifications), suggesting reduced survival in response to cell stress, which renders them less fit during the metastatic process. In the case of cleaved caspase 3, CC1-L cells show elevated level of this protein even in the absence of EPHA2 inhibitor. Other cell survival molecules including S6K1B, S6, BAD and ERK were not significantly affected, whereas AKT was modestly reduced in CC1-L cells at baseline upon ALW-II-41-27 treatment (Figure 2F and Supplementary Table 4). These data suggest that EPHA2 signaling is an important pathway necessary to maintain MC38 cell migration and survival, as previously shown in CRC patients by Dunne et al. [26].

### Cross-talk between EPHA2 receptor and CEACAM1-L in MC38 cells

**Figure 3:**
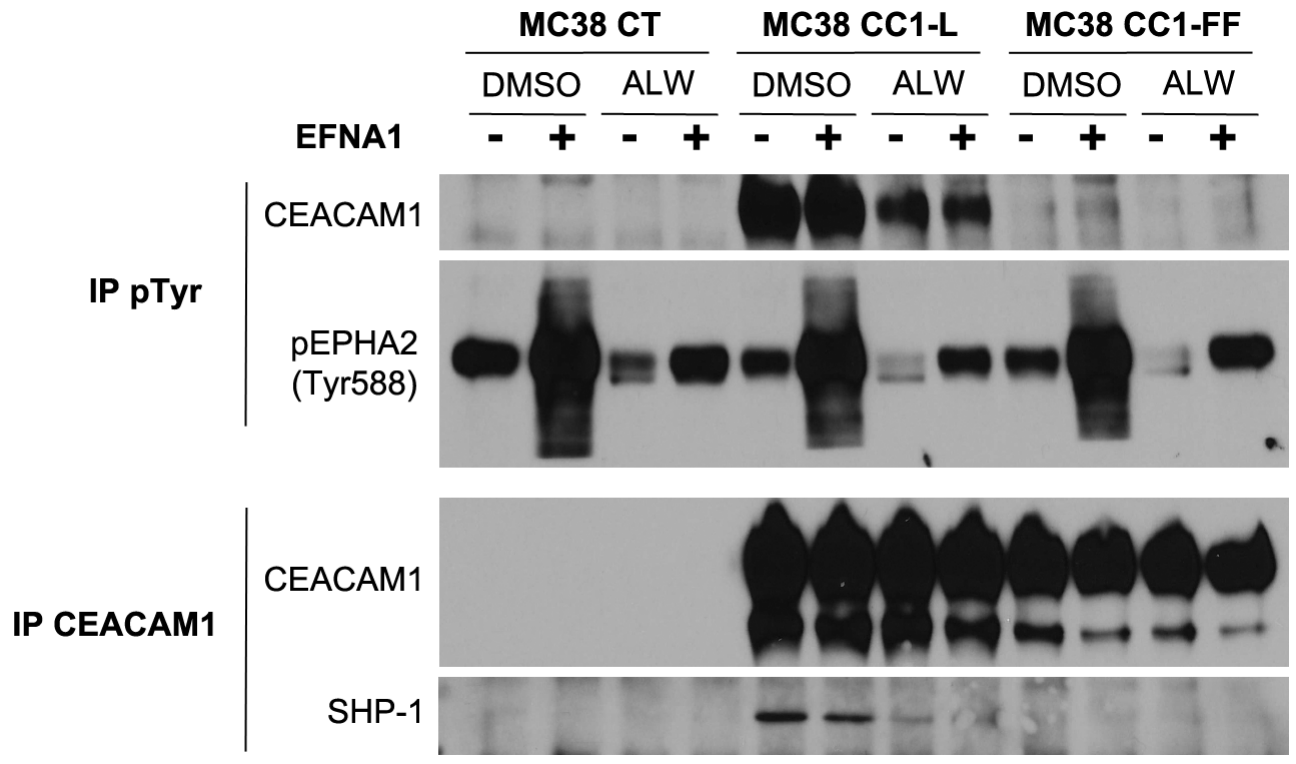
Cross-talk between EPHA2 RTK and CEACAM1 in MC38 cells MC38-CT, -CC1-L or -CC1-FF cells were treated with 1 μM DMSO or ALW-II-41-27 for 2 h, followed or not by stimulation with EFNA1-Fc (2 μg/ml) in the last 15 min of treatments. Phospho-tyrosine (pTyr) immunoprecipitates were analyzed for CEACAM1 and pEPHA2-Tyr588, while CEACAM1 immunoprecipitates were immunoblotted for CEACAM1 and SHP-1.

We next examined whether CEACAM1-L is regulating EPHA2 signaling as a co-receptor, as with many other RTKs [2]. We found no direct association of the two cell surface molecules by co-immunoprecipitation (data not shown). We then treated MC38-CT, -CC1-L and CC1-FF (CC1-L harboring mutations in both cytoplasmic ITIM-associated Tyr residues) with either ALW-II-41-27 or DMSO for 2 h, followed by brief EFNA1 stimulation. Immunoprecipitation using a pTyr antibody revealed phosphorylation of CEACAM1-L at both the basal and ligand-stimulated levels in MC38-CC1-L cells as compared to -CT cells (Figure 3). EPHA2 Tyr phosphorylation is shown as a reference. In addition, CEACAM1-L Tyr phosphorylation was significantly reduced in the presence of EPHA2 inhibitor and completely abolished in CC1-FF-mutated cells (Figure 3). Given that phosphorylated CEACAM1-L recruits the SHP-1 phosphatase in order to dephosphorylate and attenuate the signaling downstream of many RTKs [2, 45], we examined association of this phosphatase with the CEACAM1 complex. SHP-1 was present in CEACAM1 immunoprecipitates, significantly reduced upon ALW-II-41-27 treatment and absent in CEACAM1-FF immune complexes (Figure 3). These results suggest that EFNA1-mediated EPHA2 stimulation can lead to phosphorylation of CEACAM1-L ITIM-associated Tyr residues, with recruitment of its SHP-1 phosphatase partner resulting in EPHA2 downstream signaling modulation, as reported for other CEACAM1-L-mediated processes downstream other RTKs [2].

### High *CEACAM1*/low *EPHA2*/low *CEACAM6* expression predicts better outcome for CRC patients

Our experimental data indicated that CEACAM1-L expression in the context of murine MC38 CRC metastatic cells affects EPHA2 expression and signaling resulting in significantly decreased development of liver metastases in immunocompetent mice. In addition, HT29 human CRC cells abundantly express 3 of the CEACAM family members, namely CEACAM1, CEA and CEACAM6 (Figure 1B). HT29 CC1KD cells have elevated CEA and CEACAM6 levels (Figure 1B-1D) along with increased activity of EPHA2 (Supplementary Figure 3C) and produce a higher liver metastatic burden (Figure 1E). To examine whether these proteins (i.e. CEACAM1, CEA, CEACAM6 and EPHA2) are associated with clinical outcomes in CRC patient cohorts, we screened the TCGA RNA-Seq database and evaluated 514 colon and rectal tumor samples of all stages for *CEACAM1* expression in correlation with *CEACAM5 (CEA)*, *CEACAM6* and *EPHA2* over a 10-year survival period. EPHA2 displays a complex expression pattern in CRC patients, with greater expression in CRC stages 1 and 2 than in stages 3 and 4, with marked down-regulation in metastatic cases [26, 46]. EPHA2 expression acts as a driver of migration and invasion and represents a poor prognostic marker in CRC [26]. Previous studies have shown that CEA and CEACAM6 are markers of poor outcome and tumor recurrence in several types of cancers including CRC [2, 47, 48]. In addition, plasma-based CEA levels are utilized for surveillance of CRC recurrence and monitoring of disease status [49].

Using univariate (Supplementary Table 1) and multivariate (Table 1) linear regression models, we evaluated associations between the *CEACAM1*, *CEACAM5*, *CEACAM6* and *EPHA2* mRNA levels in tumors and clinicopathological parameters relative to age, gender, primary tumor site presentation, primary lymphatic presentation (i.e. a yes/no/unknown indicator whether a lymph node assessment was performed at the primary presentation of disease), lymphatic and venous invasion. In multivariate analyses (Table 1), as described in Materials and Methods, the most significant p value associations (*P* < 0.1) based on analyses of low or high expression of each mRNA in stages 3 and 4 tumors or in tumors of all stages (T1-T4) were: as single genes, *CEACAM1* expression with male patients and primary lymphatic presentation in aged patients; *CEACAM5* expression with primary lymphatic presentation in aged male and female patients; *CEACAM6* expression with female patients at the primary tumor site; and finally, *EPHA2* expression with primary tumor site and primary lymphatic presentation in aged male patients and venous invasion. When examining multiple gene associations together, *CEACAM5*, *CEACAM6* and *EPHA2* expression were significant with clinical presentation in the sigmoid colon whereas *CEACAM1*, *CEACAM5* and *EPHA2* expression, with primary lymphatic presentation in patients ≥ 60 years of age. In univariate analyses (Supplementary Table 1), *CEACAM1*, *CEACAM5* and *CEACAM6* expression showed significant association with primary tumor site, while *EPHA2* expression was associated with both lymphatic and venous invasion.

**Table 1:** *P* values for multivariate analyses of associations between clinicopathological data and expression of quantile-normalized *CEACAM1, CEACAM5, CEACAM6* and *EPHA2* genes*

Stages		*CEACAM1*		*CEACAM5*		*CEACAM6*		*EPHA2*	
		T3-T4	T1-T4	T3-T4	T1-T4	T3-T4	T1-T4	T3-T4	T1-T4
**Age**	≥60	0.227	0.247	0.138	0.282	0.205	0.455	0.893	0.688
	< 60	0.524	0.297	0.294	***0.059***	0.976	0.553	0.226	0.320
**Gender**	M	***0.078***	***0.056***	0.693	0.449	0.820	0.895	0.723	0.865
	F	0.517	0.674	0.359	0.920	***0.018***	***0.092***	0.484	0.304
**Primary tumor site**	SC	0.568	0.240	***0.036***	***0.010***	0.357	***0.049***	***0.049***	***0.013***
	R	0.201	0.133	0.704	0.659	0.438	0.319	0.208	0.271
**Primary lymphatic presentation**	≥ 60	***0.013***	***0.015***	***5×10***^***-5***^	***6×10***^***-5***^	0.233	0.202	***0.012***	***0.013***
	< 60	0.848	0.997	0.987	0.746	0.734	0.809	0.917	0.892
	M	0.348	0.381	***0.035***	***0.047***	0.262	0.268	***0.091***	***0.095***
	F	0.217	0.225	***0.076***	***0.052***	0.740	0.929	0.529	0.530
**Lymphatic invasion**		0.743	0.707	0.837	0.890	0.237	0.163	0.608	0.804
**Venous invasion**		0.782	0.256	0.786	0.299	0.436	0.210	***0.021***	***0.041***

^*^ *P* values are indicated for associations between each of the genes and the indicated covariate, either for T3-T4 stages or T1-T4 stages; statistically significant values (*P* < 0.1) are indicated in bold italics. ≥ 60 or < 60, greater/equal or lower than 60 years of age; M, male; F, female; SC, sigmoid colon; R, rectum. Primary lymphatic presentation: The yes/no/unknown indicator whether a lymph node assessment was performed at the primary presentation of disease.

**Figure 4:**
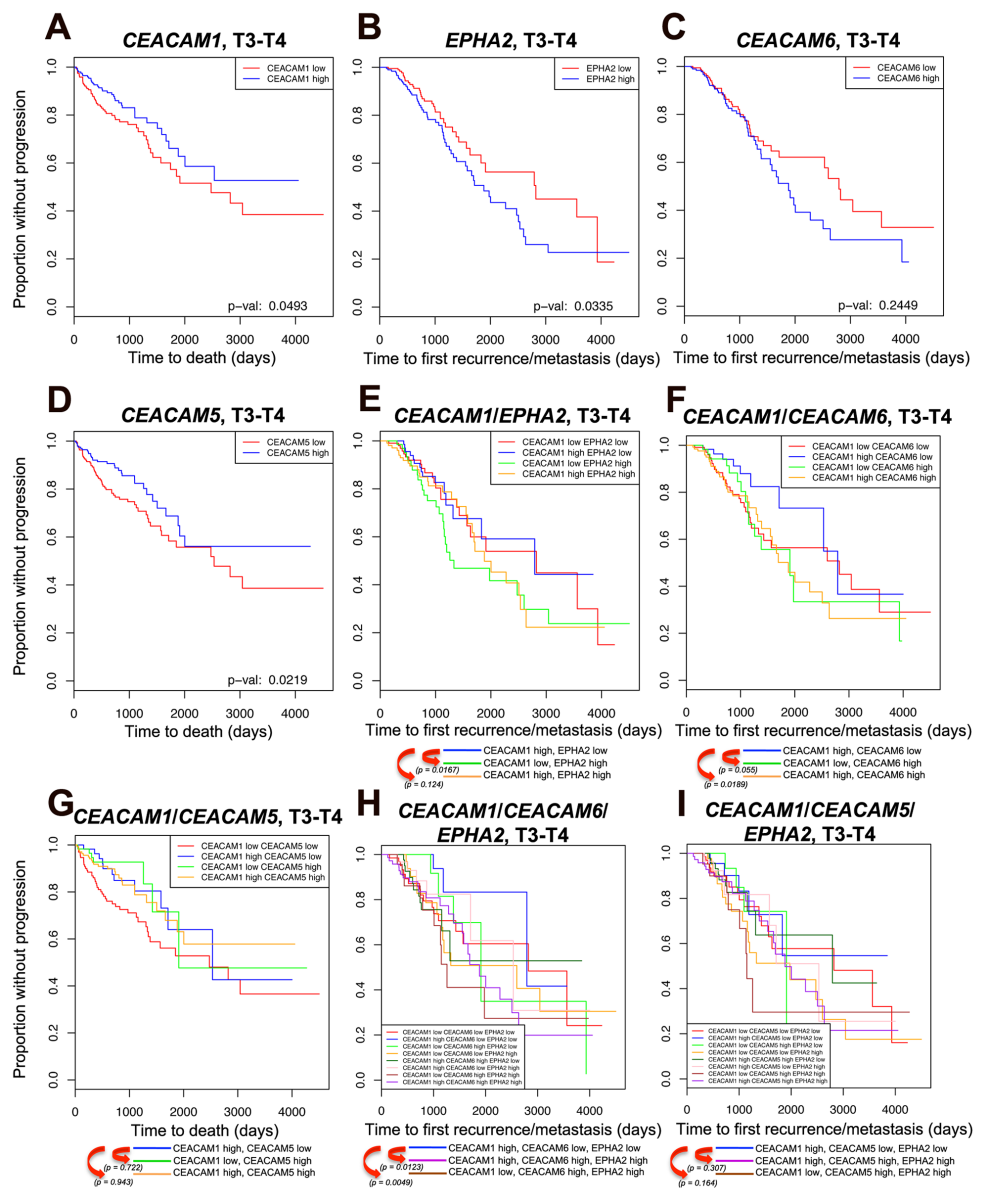
Kaplan-Meier survival analysis Kaplan-Meier survival analysis was performed on 403 colon and rectal tumor samples of T3-T4 stages examining either new metastatic tumor recurrences or death of patients enrolled in TCGA. **(A-I)** Survival data are shown for: (A) *CEACAM1*, (B) *EPHA2*, (C) *CEACAM6*, (D) *CEACAM5*, (E) combination of *CEACAM1* with *EPHA2* expression, (F) combination of *CEACAM1* with *CEACAM6* expression, (G) combination of *CEACAM1* with *CEACAM5* expression, (H) combination of *CEACAM1* with *CEACAM6* and *EPHA2* expression, (I) combination of *CEACAM1* with *CEACAM5* and *EPHA2* expression. For each gene combination, the patients were classified into high- or low-expressing groups according to whether the expression of the candidate gene was greater or smaller than the median expression of the candidate gene. The x-axis shows either time to first recurrence/metastasis or time to death in days; the y-axis shows proportion without progression. Progression is defined as metastasis recurrence or progression at the initial tumor site. For ease of comparisons in combination plots, individual *P* values have been depicted underneath the survival plots. *P* value significance, *P* < 0.1. Note that *CEA* is referred to *CEACAM5* in the new nomenclature. However, given the retention of CEA name in CEACAM research community, we have used the original name i.e. CEA throughout the current study except in the TCGA results section where CEACAM5 is the given name in the TCGA database.

We next performed survival analysis examining either new metastatic tumor recurrences or death of patients enrolled in the TCGA study (TCGA version January 2016) (Figure 4). Of 514 patients of all stages, 104 developed new metastatic lesions and were considered as “events” in the analysis (94 for T3 and T4 stages). High *CEACAM1* expression is significantly predictive of a better 10-year overall survival in stage 3 and 4 tumors (Figure 4A, *P* < 0.05) as well as in tumors of all stages (Supplementary Figure 6A, *P* < 0.1). Kaplan-Meier analyses also indicated that patients exhibiting low *EPHA2* expression in either stage 3 and 4 (Figure 4B, *P* < 0.05) or those of all stages (Supplementary Figure 6B, *P* < 0.1) fared better over a 10-year survival course in terms of time to first recurrence/metastasis, whereas the *CEACAM6* expression was not statistically indicative despite showing a similar trend (Figure 4C, *P* = 0.244 for T3 and T4 stages; Supplementary Figure 6C, *P* = 0.133 for all stages). On the other hand, high *CEACAM5* RNA expression correlated with better overall outcome (Figure 4D, *P* < 0.05 for T3 and T4 stages; Supplementary Figure 6D, *P* < 0.1 for all stages). When these same genes (*CEACAM5*, *CEACAM6* and *EPHA2)* were analyzed with the added interaction of *CEACAM1* high/low expression, we denoted that some patients with higher *CEACAM1* expression in concordance with the low expression of either *EPHA2* or *CEACAM6* exhibited better outcome than those displaying low *CEACAM1* expression and high *EPHA2* or *CEACAM6* expression (Figure 4E and 4F, blue versus green lines for T3 and T4 stages; Supplementary Figure 6E and 6F, blue versus green lines for all stages). This is reminiscent of the MC38-CT and HT29 CC1KD cells. In addition, patients exhibiting high expression of both *CEACAM1* and *CEACAM6* genes had a much poorer outcome than those with high *CEACAM1* and low *CEACAM6* expression (Figure 4F, blue versus yellow line for T3 and T4 stages; Supplementary Figure 6F, blue versus yellow line for all stages), confirming the importance of other CEACAM family members in CRC metastasis. However, the added interaction of *CEACAM5* did not reach statistical significance in any of the patient cohorts (Figure 4G and Supplementary Figure 6G).

Furthermore, combining high *CEACAM1* with low *EPHA2* and low *CEACAM6* expression showed very significant correlations whether in advanced disease stages (Figure 4H, blue line) or throughout CRC development (Supplementary Figure 6H, blue line) as compared to patients expressing high *CEACAM1*/high *CEACAM6*/high *EPHA2* (Figure 4H and Supplementary Figure 6H, purple lines) or those expressing low *CEACAM1*/ high *CEACAM6*/high *EPHA2* (Figure 4H and Supplementary Figure 6H, brown lines). Regarding *CEACAM5* combination effect, although similar trends could be observed in survival curves, the *P* values however, did not reach statistical threshold (Figure 4I and Supplementary Figure 6I) except in the case of high *CEACAM1*/low *CEACAM5*/low *EPHA2* as compared to low *CEACAM1*/high *CEACAM5*/high *EPHA2* in tumors of all stages (Supplementary Figure 6I). Results obtained with the *CEACAM1*, *EPHA2* and *CEACAM6* analysis in CRC patients therefore validate the experimental model developed with the MC38 and HT29 metastatic cell lines herein, such that high *CEACAM1* with low *EPHA2* expression along with added interaction of *CEACAM6* (in the case of human cells) in poorly differentiated CRC carcinomas constitutes a signature for significantly reduced CRC liver metastasis and overall better outcome. The lack of significant correlation between disease outcome and added expression of *CEACAM5* in patient cohorts may arise from the fact that the TCGA data are based on transcriptional measurements rather than serum CEA levels. Although several studies have shown that high serum CEA levels represent a poor outcome [49, 50], the prognostic value of CEA in cancer patients as well as its effect on tumor cell survival is widely debated (see further in the discussion section).

## DISCUSSION

Our group has recently shown that CEACAM1-L expression in murine MC38 poorly differentiated CRC cells dramatically reduced experimental liver metastasis as a consequence of diminished STAT3 activity and lessened CCL2 chemokine expression [22]. This was validated in a cohort of CRC patients demonstrating improved overall 10-year survival with high *CEACAM1*/low *STAT3* inflammatory signature expression. To better understand CEACAM1-L’s role in inhibition of CRC liver metastasis, we first examined a large panel of murine and human CRC cells expressing CEACAM1 or not. This not only enabled us to take into consideration genetic diversity of CRC cells, but also allowed us to examine involvement of other CEACAM family members in metastatic outcome. The impact of CEACAM expression on malignant tumors is still controversial and only a few studies have examined tandem tumor expression of several CEACAM molecules [51–55]. Moreover, our experimental setting benefited from use of both syngeneic immunocompetent (for murine CRC cells) and immunodeficient (for human CRC cells) mice, where immune differences can significantly impact the metastatic process. Although in this study we focused on the *KRAS* and *SMAD4* genetic mutations to categorize our CRC cell lines, [30–33, 56], we cannot disregard several other genes (e.g. *BRAF*, *PIK3CA*, *c-MET*) whose mutations can further impact the CRC patient disease outcome. Of all CRC cells tested in this study, only MC38 and HT29 produced different metastasis outcomes upon altered CEACAM1 expression, with CEACAM1-L over-expression in MC38 suppressing and CEACAM1 knockdown in HT29 enhancing liver metastasis. Although murine cells are intrinsically devoid of GPI-linked CEACAMs (e.g. CEA and CEACAM6), abundant expression of both CEA and CEACAM6 in HT29 cells and their up-regulation upon CEACAM1 knockdown suggests that co-regulation of the three molecules can cooperatively affect metastasis development. Indeed, HT29 cells accurately represent CRC patient samples with expression of all three proteins [2]. Similar data by Singer et al. showed that interplay of CEACAM1, CEACAM5 and CEACAM6 in human lung adenocarcinoma cells is critical for regulation of cell proliferation, differentiation and tumorigenicity [55].

**Figure 5:**
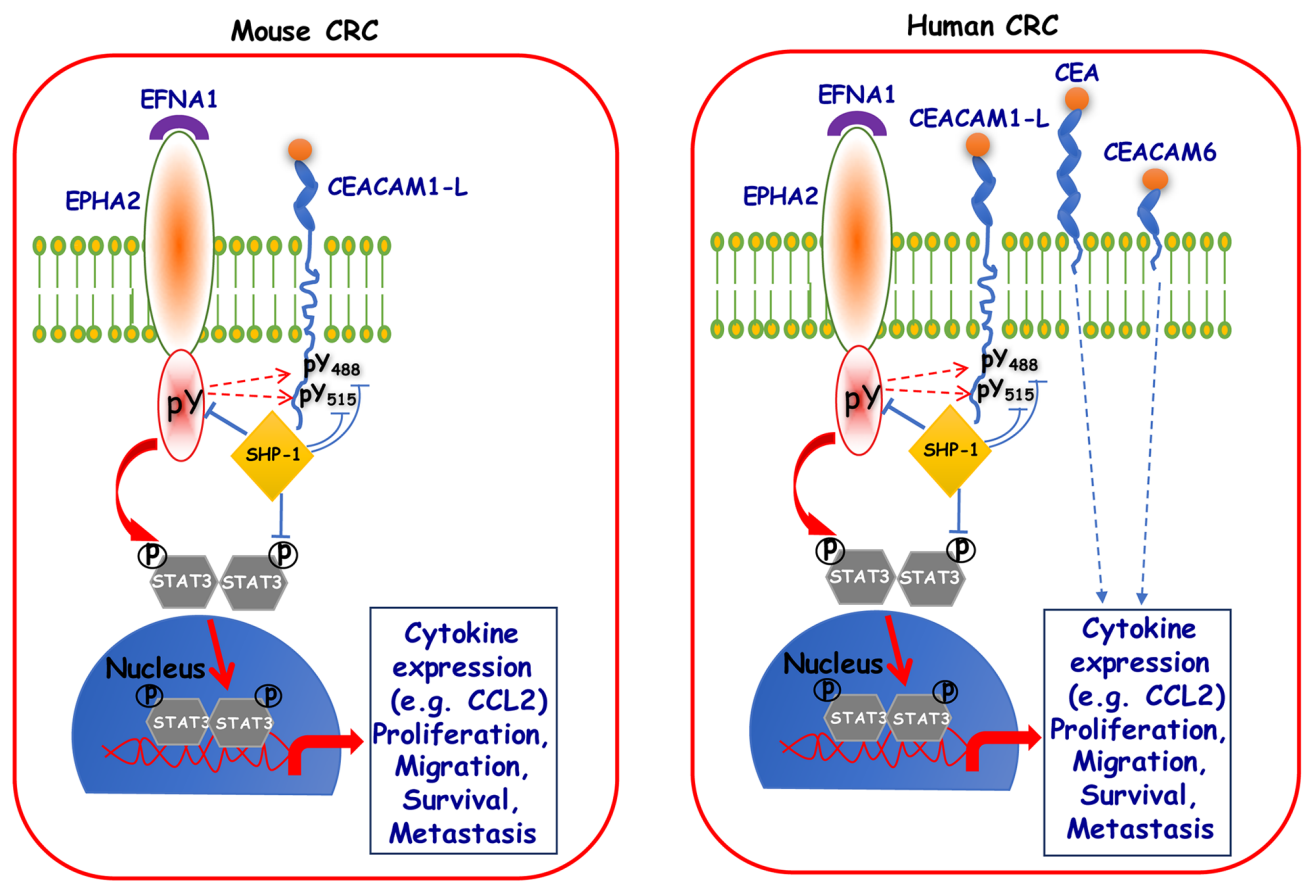
Schematic diagram depicting CEACAM1-L association with SHP-1, as a proposed mechanism for negative regulation of metastasis Several studies have demonstrated that CEACAM1-L acts as a co-receptor and a negative regulator of many receptor Tyr kinases (RTKs), thereby leading to very diverse phenotypes in different cells types. Through a series of studies from our laboratory [2, 22, 38, 49, 61, 62], we are showing that in mouse CRC cells, CEACAM1-L’s ITIM-associated pTyr moieties (488 and 515) become a docking site for recruitment of SHP-1 phosphatase, which can in turn dephosphorylate nearby RTKs (EPHA2 in the current study), negatively regulate downstream signaling and impact on metastasis. Similar scenarios can be envisaged in the case of human CRC cells, however, with the added complexity from expression of two other CEACAM family members i.e. CEA and CEACAM6.

Previously, we showed that CEACAM1-L-mediated reduction of liver metastasis is partly due to compromised STAT3 activity that results in reduced activity of CCL2-CCR2 axis [22]. Transcriptome and phospho-RTK profiling of MC38 cells have now given further insights into mechanisms of CEACAM1-mediated reduction of metastasis; we have identified that CEACAM1-L acts as a negative regulator of the EPHA2 receptor Tyr kinase, also involved in CRC progression and liver metastasis [24–26], as judged by either lower EPHA2 Tyr phosphorylation in MC38-CC1-L cells or higher pEPHA2 levels in HT29 CC1KD cells. Indeed, EFNA1 triggering of CEACAM1-L-expressing cells modified pEPHA2-Tyr588 phosphorylation, pSTAT3 dephosphorylation kinetics and pAKT activation relative to MC38-CT cells. Moreover, CEACAM1-L-expressing cells have reduced expression and activity of EPHA2, consequently exhibiting less sensitivity to inhibitory effects of ALW-II-41-27 (EPHA2 inhibitor) on proliferation and migration. EPHA2 is also a regulator of cell migration and survival, which further emphasizes implication of STAT3 and SRC signaling pathways downstream of EPHA2/CEACAM1-L, confirming our previous findings [22]. Since CEACAM1-L does not directly associate with EPHA2, the recruitment of the SHP-1 Tyr phosphatase associating with both proteins [57, 58] could constitute a possible target involved in this phenotype. This hypothesis was addressed by immunoprecipitation experiments, where EFNA1 stimulation led to CEACAM1-L Tyr phosphorylation and recruitment of SHP-1. This effect was diminished upon treatment with the EPHA2 inhibitor and completely abolished in cells expressing Tyr-mutated isoform of CEACAM1-L. This corresponds to a common theme observed with several other RTKs [2] where CEACAM1-L’s ITIM-associated pTyr moieties become docking sites for recruitment of phosphatases (including SHP-1), which in turn dephosphorylate nearby RTKs and negatively regulate downstream signaling (Figure 5).

The signaling association between CEACAM1-L and EPHA2 is further strengthened by the RNAseq data of TCGA samples indicating that patients with high *CEACAM1*/low *EPHA2* expression exhibit a more favorable 10-year survival outcome than those having low *CEACAM1*/high *EPHA2* expression. This signature becomes even more significantly representative of our experimental setting: CRC patients whose samples were collected in TCGA database with demonstrated high *CEACAM1*/low *EPHA2/*low *CEACAM6* expression benefited from a much better prognosis and longer survival. Given that EPHA2 is one of the key regulators of cell proliferation, migration, invasion and metastatic spread, targeting this RTK in several types of cancers including breast [59], CRC [24, 26, 60, 61], lung [44], brain [62] and prostate [63] impairs tumor growth and metastasis. Our results also suggest that a combined targeting approach of at least 2 targets (EPHA2 and CEACAM6) might highly improve survival of certain CRC patients. On the other hand, our result denoting correlation of high *CEACAM5* expression with better overall outcome is in contrast with high serum CEA levels representing a poor outcome prognostic marker [49, 50]. The prognostic value of CEA in cancer patients as well as its effect on tumor cell survival is widely debated. Singer et al. demonstrated that human lung adenocarcinoma cells expressing CEA have significantly reduced survival [55], while Spindler et al. showed that pre-treatment CEA plasma levels of patients with metastatic CRC treated with Cetuximab and Irinotecan were not associated with differences in overall survival or progression-free survival, while TIMP-1 showed significant prognostic value [64]. Similar controversial findings were discussed by Grunnet and Sorensen in their review of CEA use as a tumor marker in lung cancer [65]. Moreover, whether *KRAS* status or mutations of other cancer driver genes in CRC patients (TCGA database) might have led to lack of *CEACAM5* interaction with the other studied genes (*CEACAM1*, *CEACAM6* and *EPHA2*) will need further investigation.

In conclusion, the results of our study highlight several important points: they provide insight into complexity and diversity of CEACAM1, CEA and CEACAM6 expression and their functions in CRC metastasis. As schematized in Figure 5, our data have identified and validated a new signaling axis in poorly differentiated murine SMAD4-mutated and wild-type RAS CRC cells that is influenced by the CEACAM1 expression/activity. Upon activation of the EPHA2 receptor by its cognate EFNA1 ligand, CEACAM1-L and STAT3 become tyrosine phosphorylated by either the EPHA2 kinase or a SRC-like kinase activated by this receptor (as shown by dotted lines in Figure 5). Phosphorylated STAT3 translocates to the nucleus to exert its transcriptional activity, thus activating several signaling pathways resulting in heightened cytokine expression, cell proliferation, migration and invasion. Tyrosine phosphorylation of CEACAM1-L provokes the recruitment to the vicinity of the receptor and association with the tyrosine phosphatase SHP-1 that then decreases inherent tumor cell EPHA2-mediated signaling along with reduced STAT3 activity, rendering the development of *in vivo* CRC liver metastasis inefficient. Many human CRC cells also express CEA and CEACAM6 that independently influence *in vivo* CRC liver metastasis (Figure 5). The effective delivery of novel EPHA2 specific inhibitors [66] or STAT3 inhibitors [67] may thus become therapeutic modalities available for some CRC metastatic patients in the future. Finally, our proposed signature combining three genes (high *CEACAM1*/low *EPHA2*/low *CEACAM6*) can be used for screening and identifying CRC patients with better outcome.

## MATERIALS AND METHODS

### Cell lines and cell culture

Metastatic mouse MC38 and CT26 CRC cells were maintained in either D-MEM (Life Technologies, Carlsbad, California) or A-MEM (Wisent, St-Bruno, Quebec) respectively. The human CRC cells were maintained in RPMI 1640 (Life Technologies, Carlsbad, California) (for HCT116, Colo320 and KM12), A-MEM (for HT29, LS174T and LS180) or D-MEM (for SW620). All media were supplemented with 10% heat-inactivated fetal bovine serum (FBS, Life Technologies, Carlsbad, California), 100 U/ml penicillin and 10μg/ml streptomycin (Wisent, St-Bruno, Quebec). Cells were grown in a humidified incubator with 5% CO_2_ at 37°C. Stable CC1 KD human cell lines (HT29, SW620 and KM12) were generated with pLKO.1 vector (with puromycin as selection) containing the human CEACAM1 shRNA, while for CEACAM1-L stable overexpression in CC1-L lines (LS174T, LS180 and Colo320), we used PQCXIB vector (with Blasticidin as selection) containing the human CEACAM1-L cDNA. All control cell lines (CT) were generated using the same empty vectors. HT29 and KM12 cells were subjected to lentiviral infection, while the others were transfected using Lipofectamine (Life Technologies, Carlsbad, California). MC38, CT26 and HCT116 cells expressing either the murine CEACAM1-L (MC38 and CT26) or human CEACAM1-L (HCT116) or empty (CT) constructs have been reported elsewhere [68–70]. MC38 cell lines were subjected to incubation with 1 μM of either ALW-II-41-27 or NG-25 (MedChem Express, NJ; dissolved in DMSO), with 20 ng/ml of mouse epidermal growth factor (EGF) (Sigma; Saint-Louis, MO) or 2 μg/ml of EFNA1 (R & D Systems; Minneapolis, MN).

### Immunoblot analyses

Cell pellets were lysed in the buffer described [19]. Proteins were separated on SDS-PAGE gels, transferred to PVDF membranes and immunoblotted with the following antibodies (Supplementary Table 3): phospho- and total STAT3, ERK, AKT, EPHA2 (Tyr588 and Ser897), ERBB2 (Tyr1221 and Tyr877), PDGFRA, SRC, p38, S6K1B, S6, BAD, cleaved caspase3 (all from Cell Signaling, Danvers, MA), pTyr 4G10 (Millipore, Billerica, MA), SHP-1 (kind gift from Dr. A. Veillette), mouse CC1 (kind gift from Dr. K.V. Holmes), human CEACAM1 (Millipore, Billerica, MA), CEA (kind gift from Dr. C. P. Stanners), CEACAM6 (BioLegend, San Diego, CA), SMAD4 (Santa Cruz, Dallas, TX) and beta-Actin (BD Biosciences). The secondary HRP anti-rabbit or -mouse antibodies (GE Healthcare, Baie-D’Urfe, Quebec) were detected using the Western Lightning Plus-ECL kit (Perkin-Elmer, Waltham, MA).

### Immunoprecipitation

MC38 cells were treated with either 1 μM ALW-II-41-27 (MedChem Express, NJ; dissolved in DMSO) or 1 μM DMSO for 2 h, followed by EphrinA1 (R & D Systems; Minneapolis, MN; 2 μg/ml) stimulation in the last 15 min of treatments. Cells were then lysed in the buffer described [19]. 400 μg of Lysates was subjected to immunoprecipitation using either 4G10 anti-pTyr mAb, or anti-mouse CC1 mAb. Immunoprecipitates were analyzed by SDS-PAGE and immunodetection was performed as described above (Supplementary Table 3) using mouse CC1, pEPHA2 (Tyr588), and SHP-1 antibodies.

### *In vivo* experiments and metastasis induction

For mouse CRC metastasis assays, 8 - 10 week old C57BL/6 or BALB/c wild-type mice (Harlan; Montreal, Quebec) received an intrasplenic injection of 2 × 10^5^ viable MC38 or CT26 cells, respectively. For human CRC cell lines, 8 - 10 week old SCID Beige mice (Charles River Laboratories; St-Constant, Quebec) were injected intrasplenicaly with either 1 × 10^6^ (LS174T, LS180, SW620 and Colo320) or 2 × 10^6^ (HT29, HCT116 and KM12) cells. All CRC cells were resuspended in 50-100 μl of phosphate-buffered saline (PBS), injections were performed under general anesthesia and followed by splenectomy 3 min post-injections. Mice were sacrificed either 2 weeks (for mouse CRC cells) or 4-6 weeks (for human CRC cells) post-injection and tissues were collected to assess liver metastasis. All experiments were repeated twice with a minimum of 10 mice per condition.

### Ethics statement

This investigation has been conducted in accordance with the ethical standards and according to the Declaration of Helsinki and according to national and international guidelines. All experimental animal procedures were approved by the McGill University Animal Ethics committee (protocol 5008) in accordance with the guidelines of the Canadian Committee on Animal Care.

### Proliferation, migration and invasion assays

MC38-CT and -CC1-L cells were plated in 16-well E-Plates or CIM-Plates (ACEA Biosciences; San Diego, CA) and proliferation, migration and invasion were measured in real-time using an xCELLigence instrument (Roche; Basel, Switzerland) with assays performed in the presence of 1μM ALW-II-41-27 or NG-25 or DMSO. Fetal bovine serum (FBS; 10%) was used as chemoattractant in migration and invasion assays, with serum-free medium (SFM) as a negative control. Invasion assays were also performed using Matrigel-coated BD Falcon Cell Culture Inserts (BD Biosciences; Durham, NC) as described [71].

### RNA extraction, cDNA synthesis and qPCR

Total RNA was isolated using the Qiagen RNeasy mini kit (Qiagen, Toronto, ON). To quantify gene expression levels, equal amounts of cDNA were synthesized using the InVitrogen cDNA synthesis kit (InVitrogen, Carlsbad, CA) and mixed with the hot start reaction SYBR Green I PCR master mix (Roche Diagnostics, Indianapolis, IN, USA) containing 10 μM of the primers (BioCorp; Dollard-Des Ormeaux, Quebec) described in Supplementary Table 2. *Psmb6* or *RPLP* was amplified as an internal control. All qPCR reactions for mouse primers were conducted using the LightCycler^®^ 480 Instrument (Roche Life Science, Indianapolis, IN, USA) at 95°C for 5 min, followed by 45 cycles of 95°C for 10 s, 60°C for 10 s and 72°C for 10 s. Using primers for the human cDNAs, the reactions were performed at 95°C for 10 min, followed by 45 cycles of 95°C for 15 s, 60°C for 20 s and 72°C for 20 s. The specificity of the reaction was verified by melting curve analysis. The relative quantification of each mRNA was performed using the comparative Ct method. Data processing was performed using LightCycler^®^ 480 software (Roche Life Science).

### Transcriptome and phospho-receptor tyrosine kinase (RTK) screen analysis

MC38-CT and -CC1-L RNA (n = 4 independent) was prepared for microarrays studies. Hybridization of samples was performed by the Centre d’Innovation de Genome Quebec facility (Montreal, QC, Canada) on Illumina chips (MouseWG-6_V2). The quality control of each chip was performed as recommended by the lumi package [72]. A filtering step was applied based on detection of *P* (< 0.05), representing the probability of the observed signal being significantly different than the background signal (noise). We retained only probes with a detection *P* < 0.05 in 50% of replicates in each experimental group. The VST algorithm was applied to stabilize variance of the raw expression level [73] and normalization was applied with the Robust spine normalization (RSN) algorithm included in lumi package. Differential analysis of the conditions of experiment was then performed using one-way ANOVA (by limma package) and we retained only the probes with a F-value < 0.05 and FDR < 0.05 (total of 5697 probes). Further functional analyses were performed on these selected probes. Data were deposited at GEO (Gene Expression Omnibus; http://www.ncbi.nlm.nih.gov/geo/info/submission.html) under accession number GSE73208. For phospho-RTK determination, cell lysate samples of MC38-CT and -CC1-L cells were applied to mouse Phospho-Receptor Tyrosine Kinase Array (R&D Systems) according to manufacturer’s instructions. Validation of candidate genes was performed using Q-PCR analyses on RNA isolated from independent samples. These results were validated on independent samples using qPCR (see primers in Supplementary Table 2) or using total protein and phospho-protein antibodies.

### Analysis of patient samples using the Cancer Genome Atlas (TCGA)

Data were analyzed from the January 2016 version of the TCGA colon and rectal adenocarcinoma (COADREAD) dataset. When colon and rectal carcinomas were sequenced on both Illumina HiSeq & GA platforms, only the HiSeq results were used. Zero reads were replaced with a random number based on the mean and standard deviation of very low read numbers (where the number of reads is smaller than 1). Two normalization methods were used, quantile normalization of all samples, and a Z-transformation of batches with at least 10 samples. For the Z-transformation, the overall mean of gene expression in each batch was Z-transformed to have mean 0 and standard deviation of 1. This gauged if a gene was expressed at a high or low level within this specific batch, and corrected for batch-specific effects. The batch-specific Z-filtered and quantile scores were used in all further survival calculations. First recurrence or progression was defined as the minimum of the six follow-up variables, *days_to_new_tumor_event_after_initial_treatment* in the TCGA COADREAD clinical dataset (columns 458, 481, 501, 518, 525, 561). There were 514 samples with both gene expression and survival data, and of these, time to first recurrence/metastasis was available for 104, and time to death was available for 103. Only 479 (95 recurrence/metastasis events) were included in the Z-transformed analysis restricting to batches of 10 or more samples. T3, T4, T4a and T4b colorectal carcinomas were also separately analyzed which narrowed the number of samples to either 335, from which time of first recurrence/metastasis was available for 71 (Z-normalization) or 403, from which time of first recurrence/metastasis was available for 94 (Q-normalization). Results of the time to recurrence analyses should be interpreted as suggestive only, since no corrections for multiple testing were used.

### Statistical analysis

All data is expressed as mean ± SEM. Statistical analyses were performed using GraphPad Prism 6 software (GraphPad Software, Inc. La Jolla, CA, USA) or R (www.cran-r.project.org). Results were considered significant if *P ≤* 0.05 using either a Student two-tailed *t* test or one way-ANOVA and the Bonferroni post-test multiple testing correction. Multivariate linear regression models were used to examine associations between *CEACAM1* expression and other genes, correcting for age, gender, primary tumor site, primary lymphatic presentation, lymphatic invasion, and venous invasion. Survival analysis used Kaplan-Meier curves with significance set at 0.1.

### Additional information

Supplementary Information accompanies this paper.

## SUPPLEMENTARY MATERIALS FIGURES AND TABLES





## Abbreviations

(CEACAM1-L or CC1-L): Carcinoembryonic antigen cell adhesion molecule 1 long isoform
(EPHA2): ephrin type-A receptor 2
(ITIMs): immunoreceptor Tyr inhibition motifs
(GPI): glycosylphosphatidylinositol
(RTK): receptor Tyr kinase
