# Supplementary material for: EphA2 signaling is impacted by carcinoembryonic antigen cell adhesion molecule 1-L expression in colorectal cancer liver metastasis in a cell context-dependent manner
Source: Oncotarget. 2017 Nov 1;8(61):104330–46. doi: 10.18632/oncotarget.22236 (PMC5732810; doi:10.18632/oncotarget.22236)
Supplement: Supplementary file 1 [file oncotarget-08-104330-s001.pdf]

EphA2 signaling is impacted by carcinoembryonic antigen cell adhesion molecule 1-L expression in colorectal cancer liver metastasis in a cell context-dependent manner

SUPPLEMENTARY MATERIALS

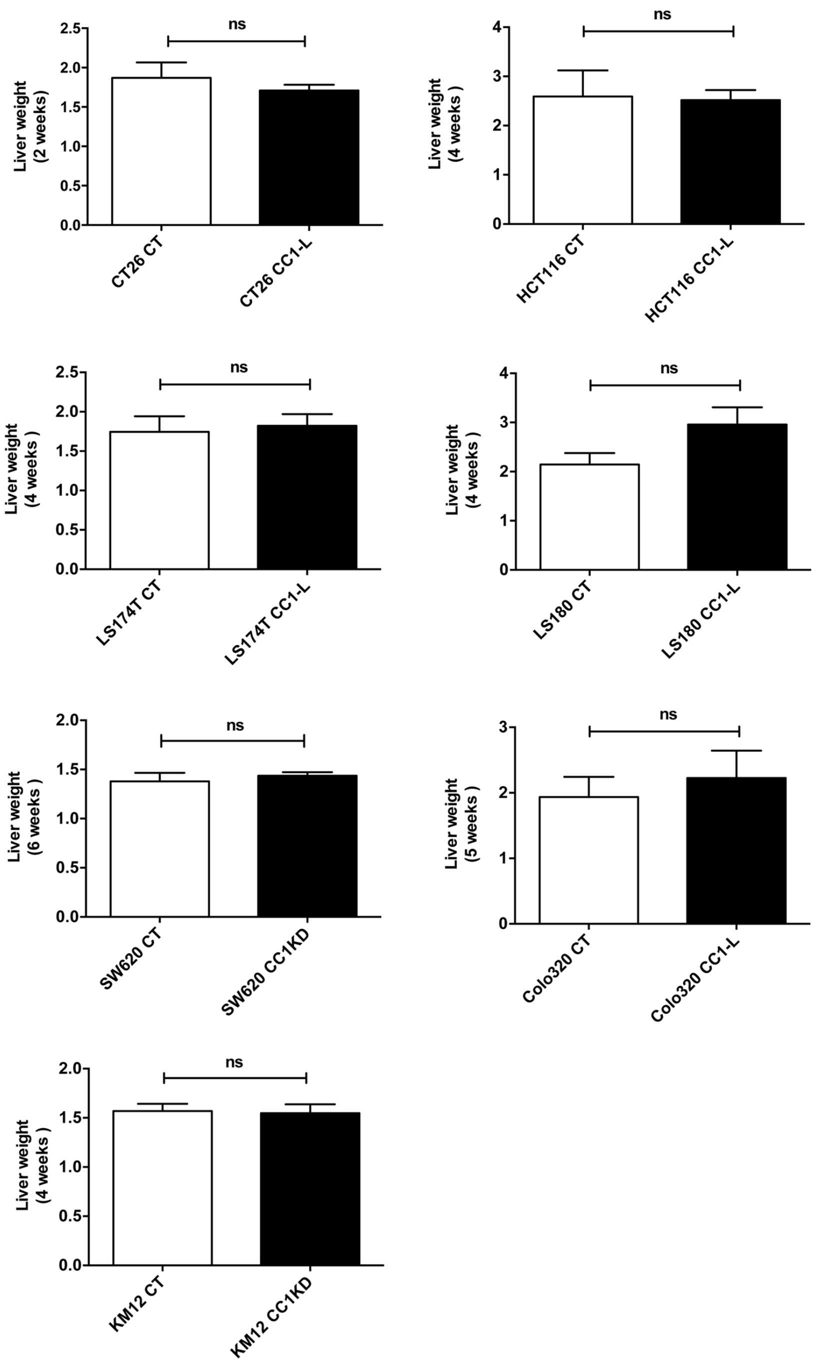

Supplementary Figure 1: Results of *in vivo* metastasis assays using mouse and human CRC cells are depicted. Mice were injected as described in Materials and Methods. Metastasis-harboring livers were dissected out at the end point of assays and weighed. Student two-tailed *t* test was performed to determine significance (ns, $P > 0.05$; *, $P < 0.05$; **, $P < 0.01$; ***, $P < 0.001$). Data are presented as means ± SEM with n = at least 2 independent sets of experiments. A minimum of 10 mice per group were used.

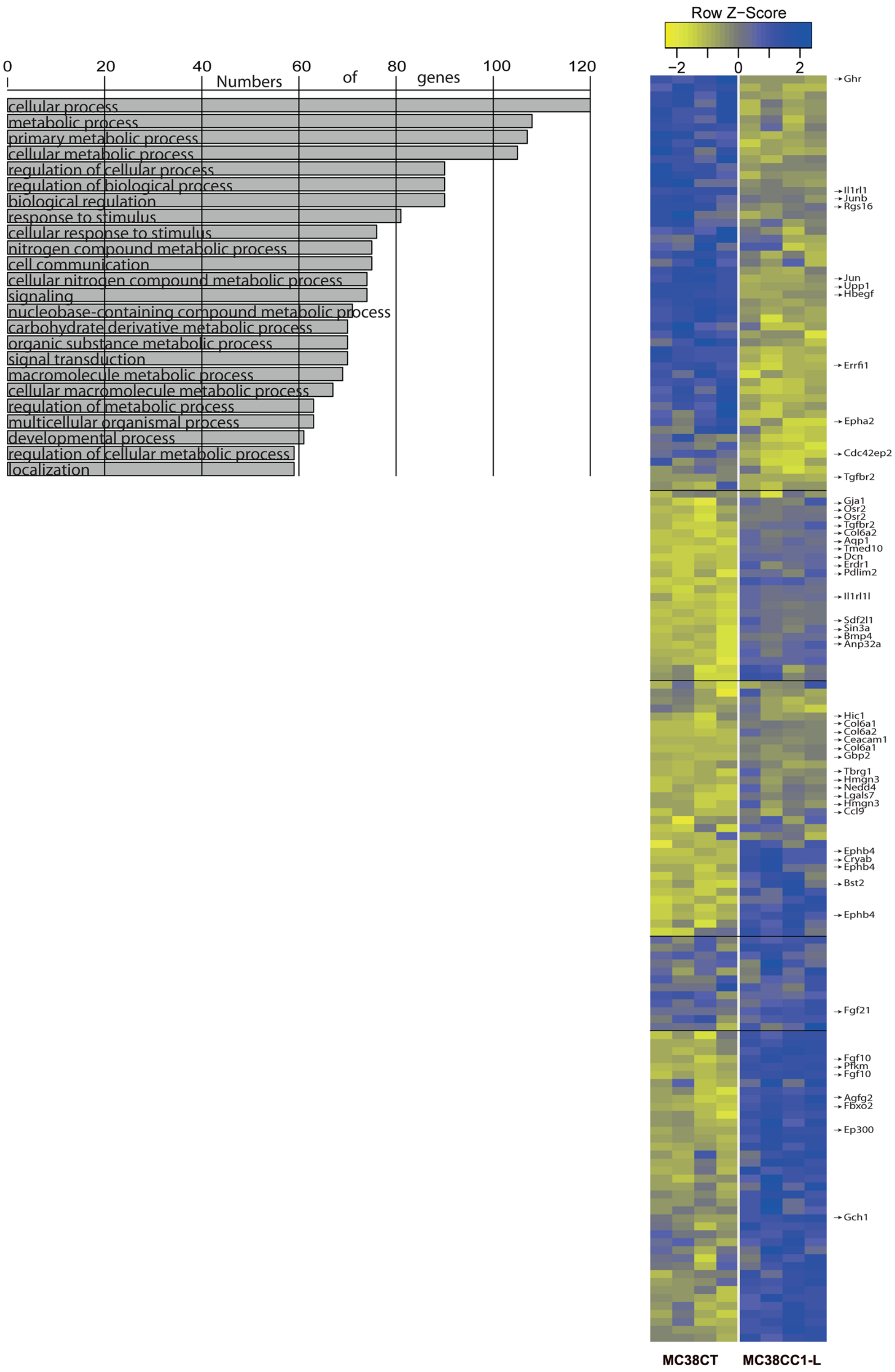

Supplementary Figure 2: Presentation of biological processes over-represented as well as heatmap of 40 selected genes in the microarray analysis of MC38- CC1-L vs -CT cells. On the heatmap, the Z-score color scale depicts level of expression of genes: intensity of blue color (0 to 2) represents overexpressed genes and that of yellow color (0 to -2) represents underexpressed genes.

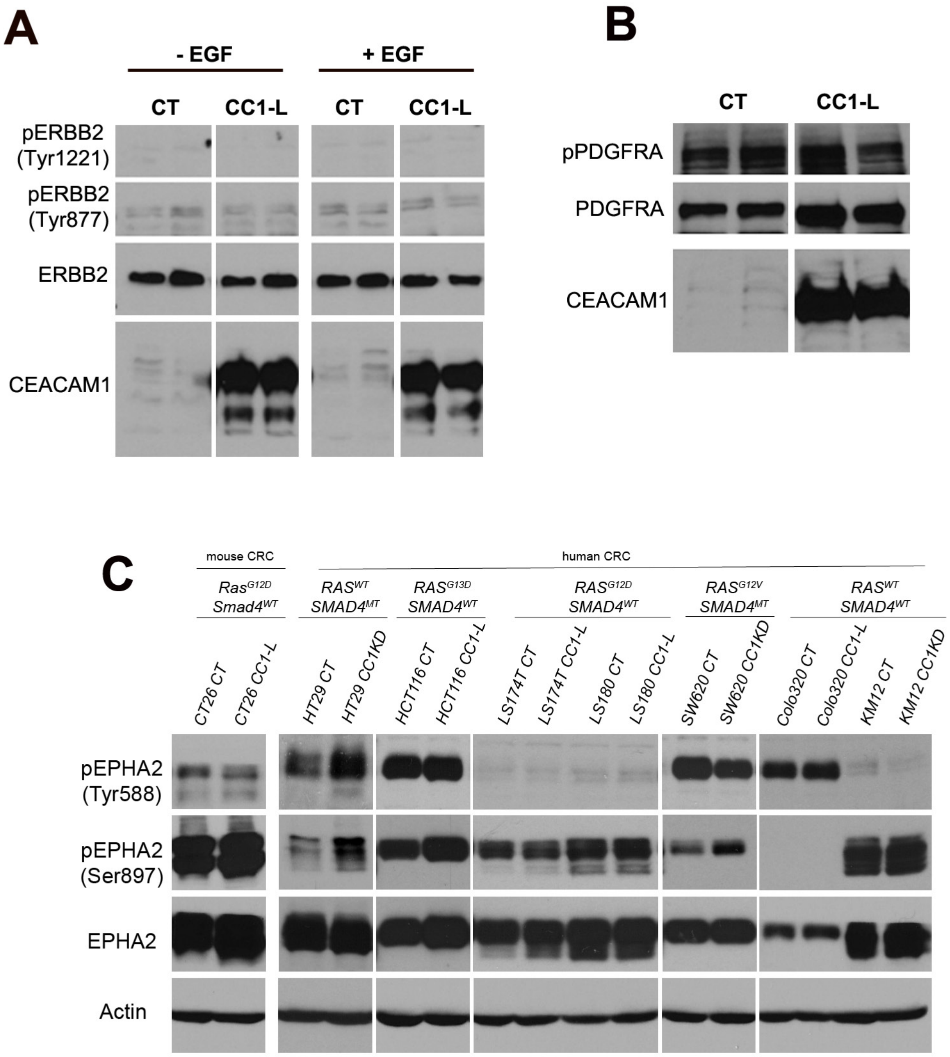

Supplementary Figure 3: (A and B) Validation of activities of two receptor tyrosine kinases (ERBB2 and PDGFRA), showing prominent levels in phosphokinase array quantification, was performed by immunoblotting of MC38-CT and -CC1-L cell lysates. Actin was used as the loading control. For ERBB2 activation (A), cells were also stimulated or not with 20 ng/ml recombinant EGF as baseline activity of this receptor tyrosine kinase is usually very low. **(C)** Evaluation of expression level and activity of EPHA2 receptor Tyr kinase in various mouse and human CRC cells. Mutational status of CRC cells relative to *KRAS* and *SMAD4* are indicated above the blots.

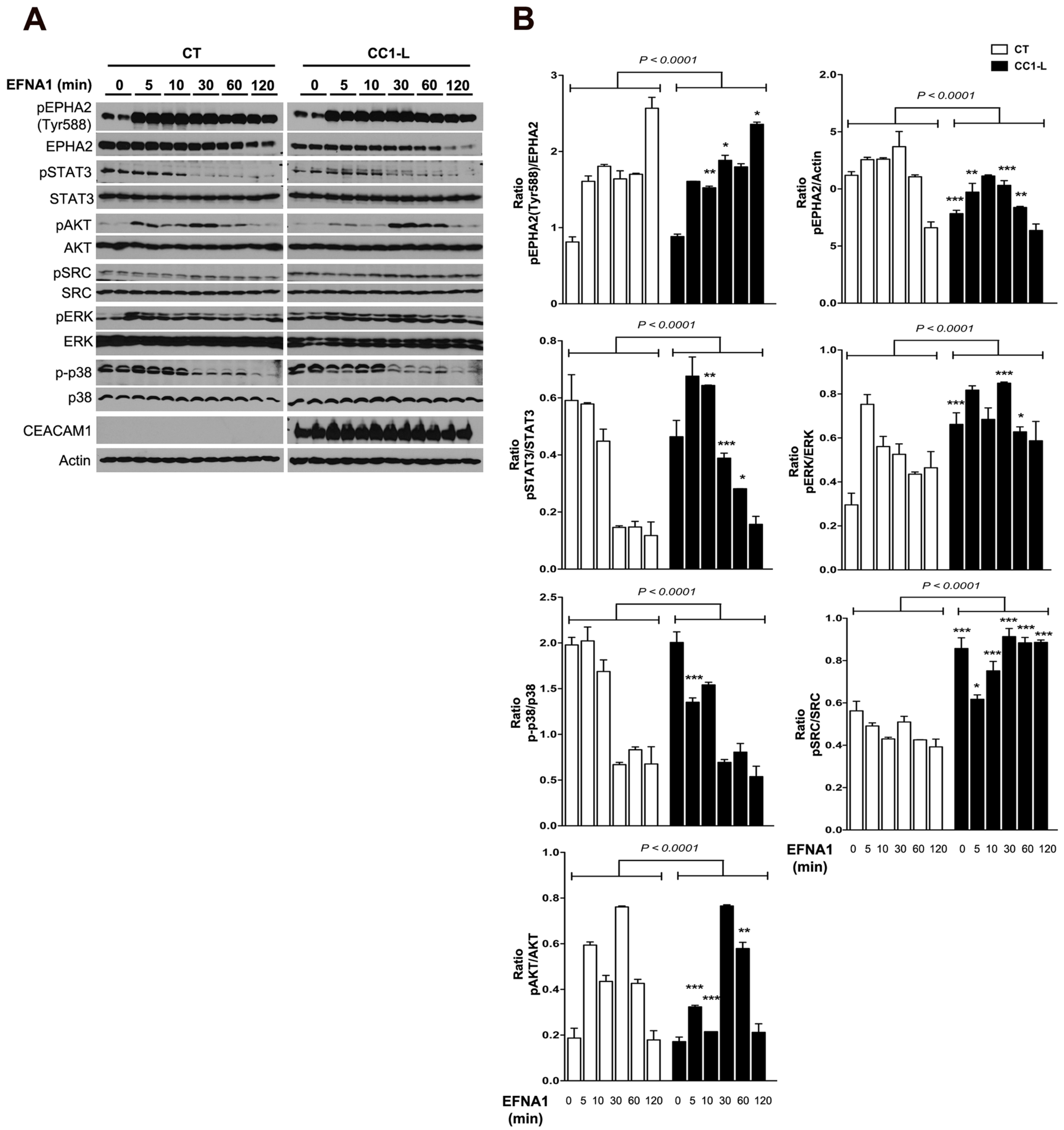

Supplementary Figure 4: (A and B) MC38-CT or -CC1-L cells were stimulated with EFNA1-Fc (2 μg/ml) for 2 h. Protein lysates were prepared at different time points and subjected to immunoblotting using various antibodies. Actin was used as the loading control. Expression levels of various signaling proteins were quantitated for n=2 separate experiments and plotted. Student two-tailed *t* or ANOVA with Bonferroni correction tests were performed as appropriate to determine significance (ns, $P > 0.05$; *, $P < 0.05$; **, $P < 0.01$; ***, $P < 0.001$). Data are presented as means ± SEM with n = at least 2 independent sets of experiments.

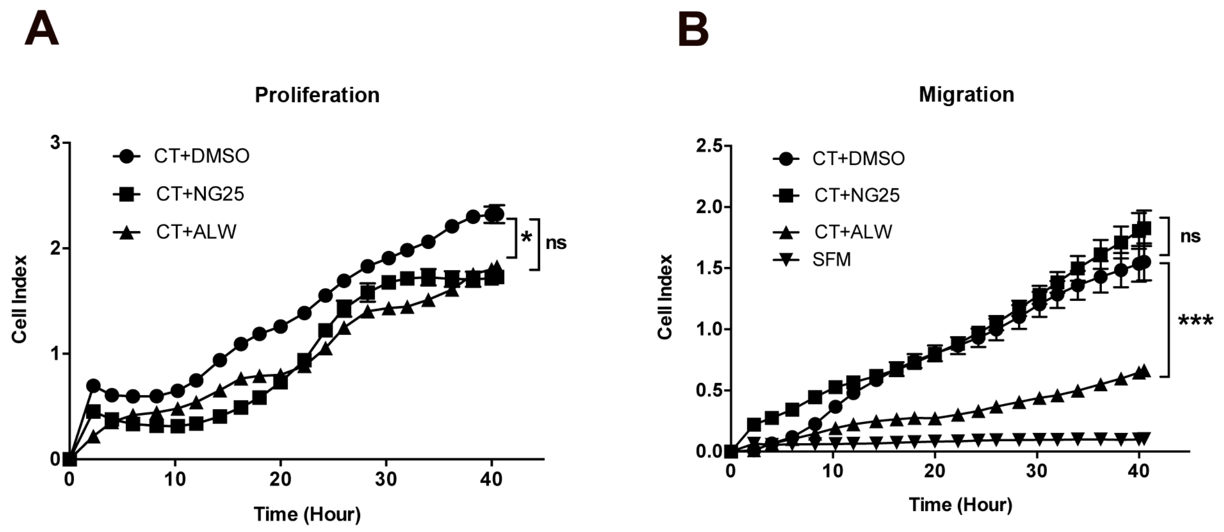

Supplementary Figure 5: Effect of two receptor Tyr kinase inhibitors, differing in their activity against EPHA2, was examined through proliferation and migration assays. MC38-CT cells were treated with 1 µM DMSO, NG-25 or ALW-II-41-27 for 2 h after which they were set up for proliferation **(A)** and migration **(B)** assays. Migration took place in the presence of 10% serum as chemoattractant, while serum-free medium (SFM) was used as negative chemotaxis control. Student two-tailed *t* or ANOVA with Bonferroni correction tests were performed as appropriate to determine significance (ns, $P > 0.05$; *, $P < 0.05$; ***, $P < 0.001$). Data are presented as means ± SEM with n = at least 2 independent sets of experiments.

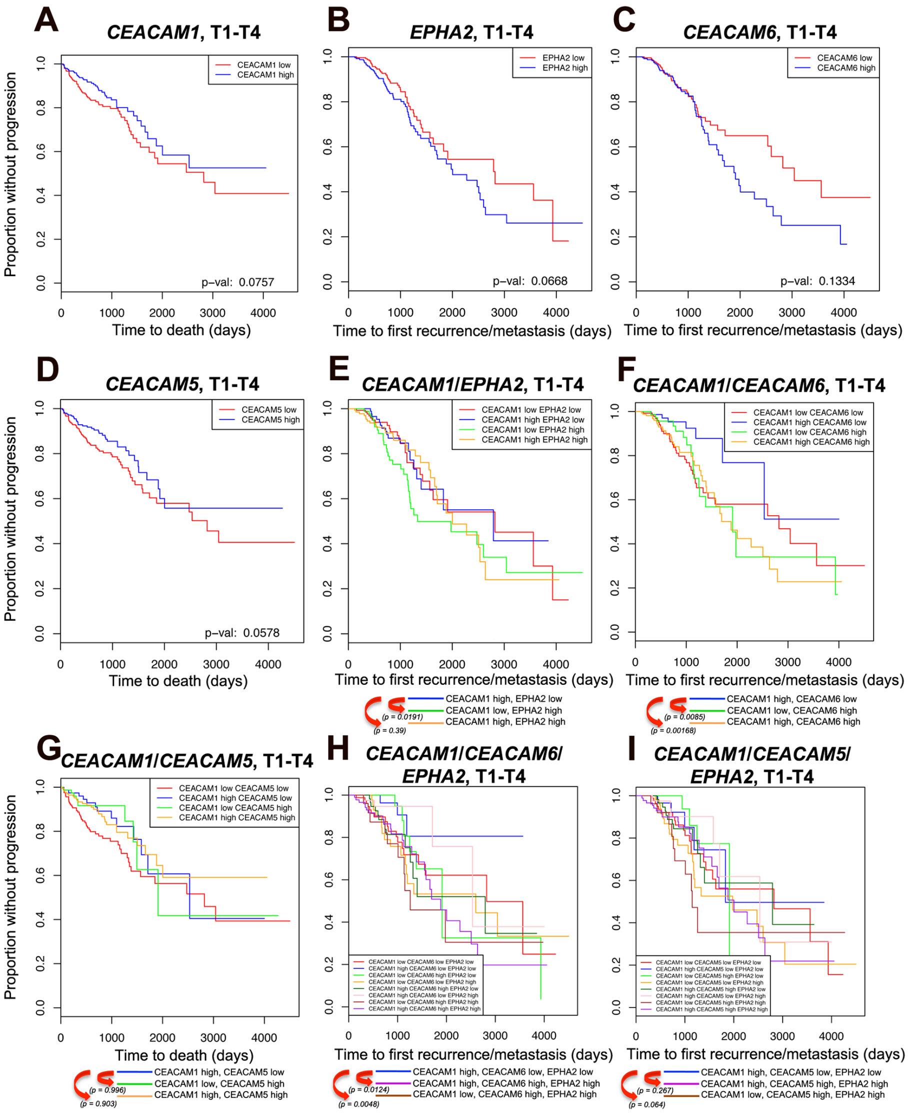

Supplementary Figure 6: Kaplan-Meier survival analysis was performed on 514 colon and rectal tumor samples of all stages (T1-T4) examining either new metastatic tumor recurrences or death of patients enrolled in TCGA. (A-I) The survival data are shown for: (A) *CEACAM1*, (B) *EPHA2*, (C) *CEACAM6*, (D) *CEACAM5*, (E) Combination of *CEACAM1* with *EPHA2* expression, (F) Combination of *CEACAM1* with *CEACAM6* expression, (G) Combination of *CEACAM1* with *CEACAM5* expression, (H) Combination of *CEACAM1* with *CEACAM6* and *EPHA2* expression, (I) Combination of *CEACAM1* with *CEACAM5* and *EPHA2* expression. For each gene combination, the patients were classified into high- or low-expressing groups according to whether the expression of the candidate gene was greater or smaller than the median expression of the candidate gene. The x-axis shows either time to first recurrence/metastasis or time to death in days; the y-axis shows proportion without progression. Progression is defined as metastasis recurrence or progression at the initial tumor site. For ease of comparisons in combination plots, individual *P* values have been depicted underneath the survival plots. *P* value significance, $P < 0.1$. Note that the *CEA* gene is referred to *CEACAM5* in the new nomenclature. However, given the retention of the CEA name in CEACAM research community, we have used the original name i.e. CEA throughout the current study except in the TCGA results section where CEACAM5 is the given name in TCGA database.

Supplementary Table 1: *P* values for univariate analyses of associations between clinicopathological data and expression of quantile-normalized *CEACAM1*, *CEACAM5*, *CEACAM6* and *EPHA2* genes[*]

	CEACAM1		*CEACAM5*		*CEACAM6*		*EPHA2*	
	**T3-T4**	**T1-T4**	**T3-T4**	**T1-T4**	**T3-T4**	**T1-T4**	**T3-T4**	**T1-T4**
**Age**	0.466	0.549	0.385	0.939	***0.060***	0.115	0.735	0.863
**Gender**	0.559	0.627	0.179	0.395	0.411	0.306	0.656	0.515
**Primary tumor site**	0.106	***0.066***	***0.002***	***0.003***	***0.0003***	***0.00013***	0.517	0.988
**Primary lymphatic presentation**	0.554	0.575	0.515	0.493	0.168	0.146	0.266	0.286
**Lymphatic invasion**	0.791	0.534	0.636	0.138	0.604	0.673	***0.009***	***0.029***
**Venous invasion**	0.617	0.218	0.424	0.085	0.642	0.563	***0.001***	***0.006***

[*] *P* values are indicated for associations between each of the genes and the indicated covariate, either for T3-T4 stages or T1-T4 stages; statistically significant values ($P < 0.1$) are indicated in bold italics. Primary lymphatic presentation: The yes/no/unknown indicator whether a lymph node assessment was performed at the primary presentation of disease.

Supplementary Table 2: Primer sequences used for qPCR experiments in this study

Gene	Forward primer	Reverse primer
Epha2	ACCAGGCTGTACTCAAGTTTAC	GCCTTCAGCGTCCCTTTAT
CEACAM5[*]	AGGCCAATAACTCAGCCAGT	GGGTTTGGAGTTGTTGCTGG
CEACAM6[*]	TCAGCCACTGGCCTCAATAG	TCTGGTCCAATCTGCCAGTC
RPLP	CGAAATGTTTCATTGTGGGAG	CATTCCCCCGGATATGAGGCAGCA
Psmb6	AGGAATCATCATTGCAGGCTGGGA	AAAGCGAGAGCATTGGCAGTGAAC

[*] Primer sequences obtained from: *Klaile et al. 2013, Respiratory Research.*

Supplementary Table 3: Antibodies used for immunodetection/immunoprecipitation in this study

See Supplementary File 1

Supplementary Table 4: Quantification of certain signaling proteins after immunodetection (results correspond to Figure 2F)

	Ratio (pSTAT3/ STAT3)	% change (upon EPHA2 inhibition)	Ratio (pSRC/ SRC)	% change (upon EPHA2 inhibition)	Ratio (p-p38/ p38)	% change (upon EPHA2 inhibition)	Ratio (cleaved caspase3/ Actin)	% change (upon EPHA2 inhibition)	Ratio (pAKT/ AKT)	% change (upon EPHA2 inhibition)
**MC38 CT (DMSO/ no serum)**	0.83	No serum: 49% reduction; Serum: 50% reduction	1	No serum: 19% reduction; Serum: 24% reduction	0.03	No serum: 5700% increase; Serum: 414% increase	0.04	No serum: 1900% increase; Serum: 1300% increase	0.06	No serum: 0% reduction; Serum: 5% reduction
**MC38 CT (DMSO/ serum)**	0.94		0.92		0.35		0.06		1.05	
**MC38 CT (ALW/ no serum)**	0.42		0.81		1.74		0.8		0.06	
**MC38 CT (ALW/ serum)**	0.47		0.7		1.8		0.84		0.99	
**MC38 CC1-L (DMSO/ no serum)**	0.43	No serum: 67% reduction; Serum: 72% reduction	0.98	No serum: 77% reduction; Serum: 76% reduction	0.04	No serum: 6400% increase; Serum: 670% increase	0.59	*No serum: 98% increase; Serum: 145% increase	0.16	No serum: 87% reduction; Serum: 12% reduction
**MC38 CC1-L (DMSO/ serum)**	0.55		0.84		0.41		0.4		0.96	
**MC38 CC1-L (ALW/ no serum)**	0.14		0.22		2.6		1.17		0.02	
**MC38 CC1-L (ALW/ serum)**	0.15		0.2		3.16		0.98		0.84	

* As compared to CT cells, CC1-L cells show elevated levels of cleaved caspase3 in the absence of EPHA2 inhibitor, therefore the further % change upon EPHA2 inhibition does not seem as pronounced as that in CT cells.
