# Supplementary material for: EphA2 signaling is impacted by carcinoembryonic antigen cell adhesion molecule 1-L expression in colorectal cancer liver metastasis in a cell context-dependent manner
Source: Oncotarget. 2017 Nov 1;8(61):104330–46. doi: 10.18632/oncotarget.22236 (PMC5732810; doi:10.18632/oncotarget.22236)
Supplement: Supplementary file 2 [file oncotarget-08-104330-s002.docx]

Supplementary Table 3. Antibodies used for immunodetection/immunoprecipitation in this study

| **Antibody** | **Type** | **Clone name** | **Specificity** | **Source** |
| --- | --- | --- | --- | --- |
| pSTAT3 | Rabbit mAb | EP2147Y | STAT3 phosphorylated at Tyr705 | Millipore |
| STAT3 | Rabbit pAb |  | STAT3 | Cell Signaling |
| pERK | Rabbit mAb | D13.14.4E | ERK phosphorylated at Thr202/Tyr204 | Cell Signaling |
| ERK | Rabbit mAb | 137F5 | C-term of p44MAP kinase | Cell Signaling |
| pAKT | Rabbit mAb | D9E | AKT phosphorylated at Ser473 | Cell Signaling |
| AKT | Rabbit mAb | C67E7 | C-term of Akt | Cell Signaling |
| pEPHA2 (Tyr588) | Rabbit mAb | D7X2L | EPHA2 phosphorylated at Tyr588 | Cell Signaling |
| pEPHA2 (Ser897) | Rabbit mAb | D9A1 | EPHA2 phosphorylated at Ser897 | Cell Signaling |
| EPHA2 | Rabbit mAb | D4A2 | EPHA2 | Cell Signaling |
| pERBB2 (Tyr1221) | Rabbit mAb | 6B12 | ERBB2 phosphorylated at Tyr 1221/1222 | Cell Signaling |
| pERBB2 (Tyr877) | Rabbit pAb |  | ERBB2 phosphorylated at Tyr 877. The antibody may cross-react with other ERBB family members (e.g., EGF receptor) when phosphorylated at their tyrosine residue | Cell Signaling |
| ERBB2 | Rabbit mAb | 29D8 | ERBB2 | Cell Signaling |
| pPDGFRA | Rabbit mAb | C43E9 | PDGF receptor α and β only when phosphorylated on Tyr849 of PDGFRα and Tyr857 of PDGFRβ | Cell Signaling |
| PDGFRA | Rabbit pAb |  | PDGF receptor a. This antibody may cross-react with PDGF receptor β. | Cell Signaling |
| pSRC | Rabbit pAb |  | SRC phosphorylated at Tyr416 may cross-react with other SRC family members (LYN, FYN, LCK, YES and HCK) when phosphorylated at equivalent sites. It does not cross-react with SRC phosphorylated at tyrosine 527. It may cross-react with phosphorylated RTKs | Cell Signaling |
| SRC | Mouse mAb | GD11 | pp60SRC | Millipore (Upstate) |
| p-p38 | Rabbit pAb |  | p38 phosphorylated at Thr180/Tyr182 | Cell Signaling |
| p38 | Rabbit pAb |  | p38-α, -β, -γ MAPK | Cell Signaling |
| pS6K1B | Rabbit pAb |  | p70 S6 kinase phosphorylated at Thr389 | Cell Signaling |
| S6K1B | Rabbit pAb |  | p70 S6 kinase protein, also recognizes p85 S6 kinase | Cell Signaling |
| pS6 | Rabbit mAb | D57.2.2E | S6 ribosomal protein phosphorylated at Ser235 and 236 | Cell Signaling |
| S6 | Mouse mAb | 54D2 | S6 ribosomal protein | Cell Signaling |
| pBAD | Rabbit pAb |  | BAD phosphorylated ar Ser112 | Cell Signaling |
| BAD | Rabbit pAb |  | BAD | Cell Signaling |
| cleaved caspase3 | Rabbit pAb |  | The large fragment (17/19 kDa) of activated caspase-3 resulting from cleavage adjacent to Asp175. This antibody does not recognize full length caspase-3 or other cleaved caspases | Cell Signaling |
| pTyr | Mouse mAb | 4G10 | pTyr (mouse and human) | Millipore |
| SHP-1 | Rabbit pAb |  | SHP-1 (mouse and human) | Gift from Dr. A. Veillette |
| mouse CEACAM1 | Mouse mAb |  | Mouse CEACAM1 | Gift from Dr. K.V. Holmes |
| human CEACAM1 | Mouse mAb | 4D1/C2 | Human CEACAM1 | Millipore |
| CEA | Mouse mAb | A20 | CEA; Human CEACAM1 (distinguishable by difference in band size) | Gift from Dr. C. P. Stanners |
| CEACAM6 | Mouse mAb | 9A6 | CEACAM6 | BioLegend |
| SMAD4 | Mouse mAb | B-8 | SMAD4 | Santa Cruz |
| Actin | Mouse mAb | C4 | Actin (mouse and human) | BD Biosci. |
